# Small Bowel Volvulus With Chylous Ascites Following Total Gastrectomy

**DOI:** 10.7759/cureus.80384

**Published:** 2025-03-11

**Authors:** Takamichi Nishida, Daisuke Muroya, Hisaaki Shimokobe, Shin Sasaki, Satoshi Taniwaki

**Affiliations:** 1 Surgery, Tobata Kyoritsu Hospital, Kitakyusyu, JPN; 2 Emergency, Kitakyusyu City Hospital, Kitakyusyu, JPN

**Keywords:** chylous ascites, curved planar reconstruction, small bowel necrosis, small bowel volvulus, total gastrectomy

## Abstract

Torsion of the small bowel around its mesenteric axis can lead to small bowel volvulus (SBV) and subsequent small bowel necrosis. Chylous ascites (CA) is attributed to lymphatic damage and compression/obstruction of lymphatic vessels. There are few case reports of SBV with CA.

A 70-year-old man, who had a history of laparoscopic total gastrectomy 10 years ago, presented to our emergency department with acute abdominal pain that lasted for 1 hour after eating dinner. The patient was diagnosed with acute small bowel obstruction secondary to SBV based on curved planar reconstruction (CPR) and underwent emergency surgery. The abdominal cavity had CA, the small bowel and mesentery showed overall white edematous changes, and the small bowel mesentery was twisted 360 degrees, but there was no small bowel ischemia or necrosis. After detorsion, surgery was completed. The postoperative course was uneventful, and the patient was discharged on postoperative day 9.

CPR is useful for diagnosing SBV in cases without typical CT findings. SBV with CA may not require small bowel resection due to necrosis, because occluded lymphatic vessels lead to small bowel and mesenteric edema, which inhibits further twisting. Additionally, surgical intervention is performed because of acute abdominal pain. We assume that dietary guidance is important because dietary factors may also be involved in SBV.

## Introduction

Torsion of the small bowel around its mesenteric axis can lead to small bowel volvulus (SBV) and subsequent bowel necrosis. Adult SBV can be classified as primary or secondary according to the cause [1]. The mechanism of primary SBV is poorly understood, but the contributing factors may include hypermobility of the small bowel and mesentery. Secondary SBV occurs due to postoperative adhesion, tumors, diverticular disease, and bands [1]. Chylous ascites (CA) is caused by damage to the lymphatic vessels due to abdominal surgery or blunt abdominal trauma, or by compression/obstruction of the lymphatic vessels due to tumors or torsion [2]. There are few case reports of SBV with CA. We report a case of acute small bowel obstruction secondary to SBV with CA following total gastrectomy.

## Case presentation

**Figure 1:**
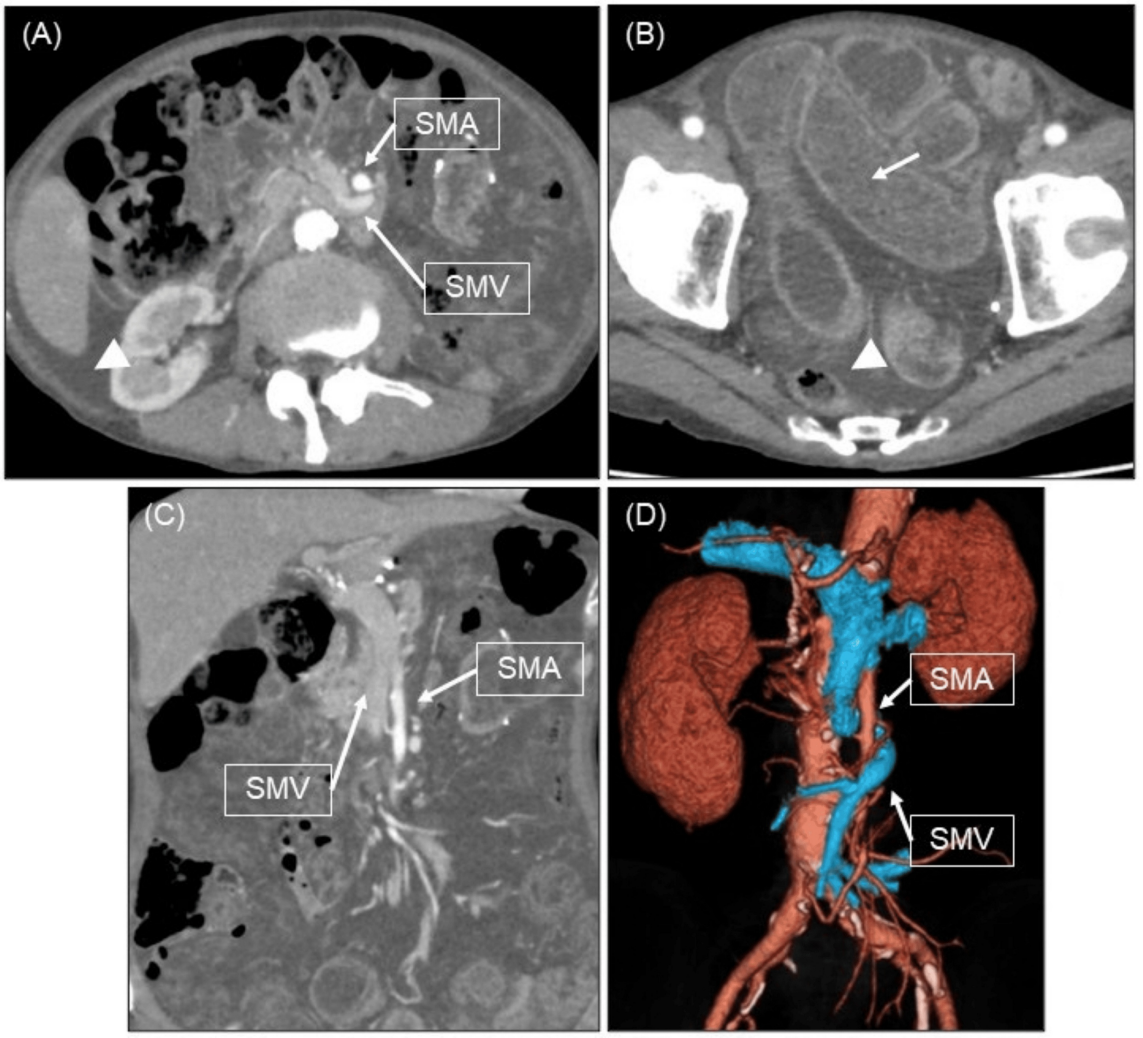
Contrast-enhanced CT and the CPR (A, B) Contrast-enhanced CT shows small bowel dilatation (white arrow) and ascites (white arrowhead). (C) Coronal section of contrast-enhanced CT. (D) The CPR definitively demonstrates a 360-degree torsion of the SMV around the main trunk of the SMA. CPR, curved planar reconstruction; SMV, superior mesenteric vein; SMA, superior mesenteric artery.

A 70-year-old man, who had a history of laparoscopic total gastrectomy, splenectomy, and Roux-en-Y reconstruction for stomach cancer 10 years ago, presented to our emergency department with acute abdominal pain that lasted for 1 hour after eating dinner. Physical examination revealed abdominal distention and tenderness throughout the abdomen with scarring from laparoscopic surgery. There were no signs of peritoneal irritation. Contrast-enhanced computed tomography (CT) showed small bowel dilatation, edematous changes in the small bowel and mesentery, and ascites, but not heterogeneous contrast effects in the small bowel wall (Figure 1A, 1B, 1C). Additionally, curved planar reconstruction (CPR) definitively demonstrated a 360-degree torsion of the superior mesenteric vein (SMV) around the main trunk of the superior mesenteric artery (SMA) (Figure 1D). The patient was diagnosed with SBV and underwent emergency surgery.

**Figure 2:**
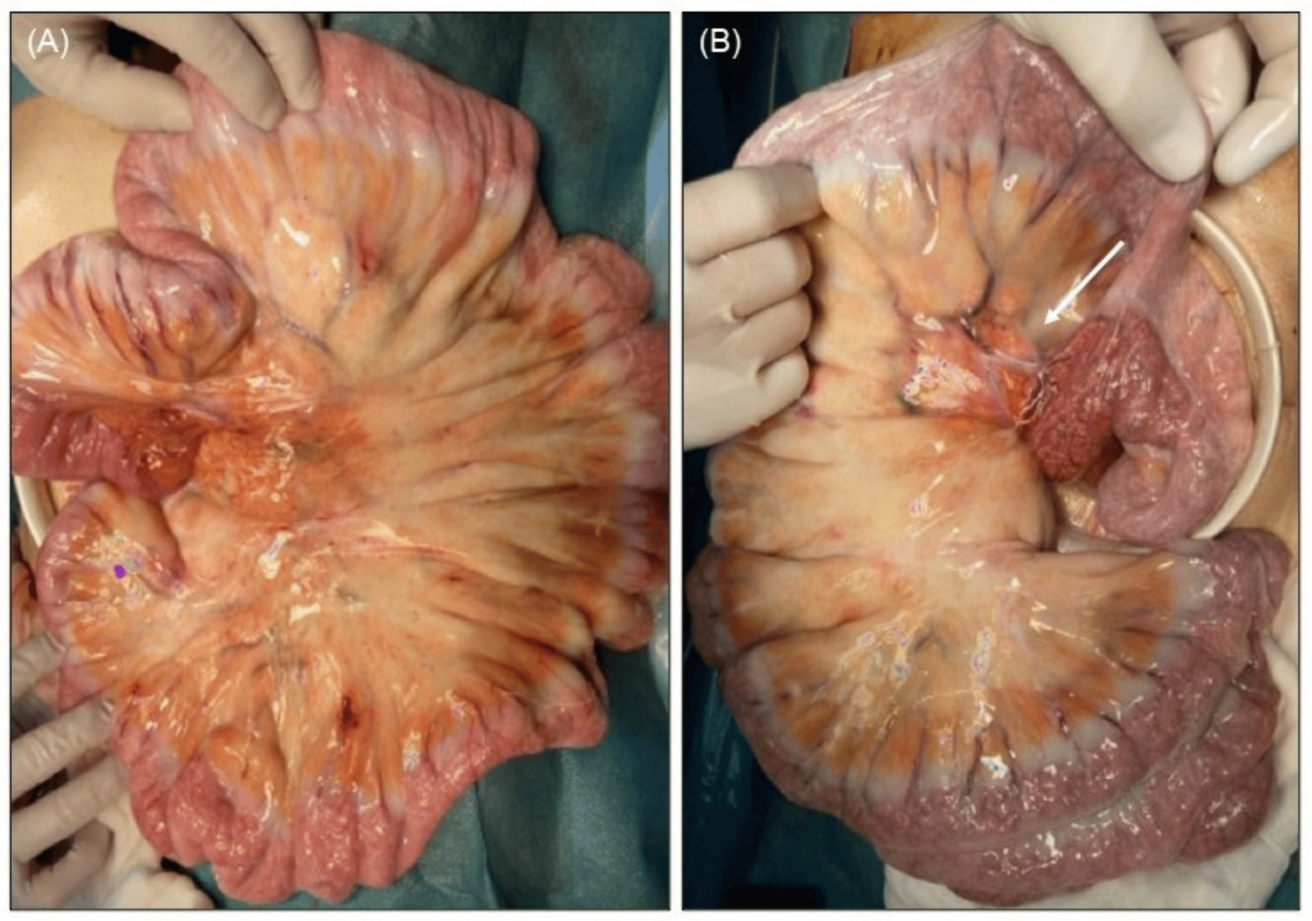
Operative findings (A) The small bowel and mesentery showed overall white edematous changes. (B) There was no anatomical abnormality in the Y-loop (white arrow) that could have caused the torsion.

A 15 cm midline longitudinal incision was made from the upper abdomen to below the umbilicus, followed by a laparotomy. The abdominal cavity had milky white ascites, but not adhesions and bands. The small bowel and mesentery showed overall white edematous changes (Figure 2A). When the small bowel was guided out of the abdominal cavity, the small bowel mesentery was twisted 360 degrees. After detorsion, observation of the small bowel and colon showed no ischemia or necrosis. Observation of the Y-loop revealed no anatomical abnormality that could have caused the torsion (Figure 2B), and the surgery was completed.

The postoperative course was uneventful, and the patient was discharged on postoperative day 9.

## Discussion

Primary SBV is also associated with dietary habits such as ingesting a large amount of bulky food at long intervals [1]. Our case had breakfast, but not lunch and presented with sudden abdominal pain after eating twice the usual amount of a curry dish for dinner. Due to a history of total gastrectomy, the sudden intake of a large amount of bulky food likely caused rapid transit into the jejunum, contributing to the development of SBV. Additionally, our case had a low BMI of 15.3 kg/cm^2^ and little visceral fat, causing a higher likelihood of hypermobility of the small bowel. We assumed that the cause of SBV in our case was a history of total gastrectomy, low body weight, and dietary factors because of no previous postoperative adhesions and bands. CA is defined as milky white ascites with a triglyceride concentration of greater than 200 mg/dL [2]. Our case was diagnosed with CA due to SBV because an analysis of the ascites showed a high triglyceride concentration of 312 mg/dL and no evidence of malignancy. The reported mechanism of CA due to SBV is that the torsion does not completely occlude the high-pressure SMA and SMV but completely occludes the low-pressure lymphatic vessels, causing lymphatic fluid to leak into the abdominal cavity [2]. SBV without CA may occur after ingesting a non-fat-rich meal or due to a lack of occlusion of the major lymphatic vessels.

We searched PubMed (National Center for Biotechnology Information, National Institutes of Health, Bethesda, MD, USA) using the keywords "adult", "chylous ascites", and "small bowel volvulus" to extract articles published from 2000 to 14 February 2025. This search retrieved the previously reported 11 cases of SBV with CA (Table 1) [2-11]. The whirl sign is a characteristic indication of SBV on CT [2]. Our case did not show the typical CT findings of SBV, probably because of the patient's low percentage of visceral fat. A whirl sign was not detected in two out of 11 cases [3,4]. In those cases, the diagnosis of SBV was not made preoperatively. Before surgery, we created CPR on the SYNAPSE VINCENT (Fujifilm Medical Corporation, Chiyoda-ku, Tokyo). CPR is a technique used to generate a reconstructed image of a curved surface along an arbitrary trajectory in three-dimensional space. This method is particularly effective for visualizing elongated and tortuous tubular structures, such as blood vessels, as well as anatomical features along the curved surfaces. In our case, CPR clearly demonstrated a 360-degree torsion of the SMV around the main trunk of the SMA. This detailed visualization allowed for the definitive diagnosis of acute small bowel obstruction caused by SBV. We suggest that CPR is useful for diagnosing SBV in cases without typical CT findings.

**Table 1:** The previously reported 11 cases and our case of adult SBV with CA M, male; F, female; LDG, laparoscopic distal gastrectomy; LTG, laparoscopic total gastrectomy; SBV, small bowel volvulus; CA, chylous ascites; PJBGC, gastric clipping with proximal jejunal bypass; LPEC, laparoscopic percutaneous extraperitoneal closure; MGBS, mini gastric bypass surgery. *Small bowel resection, including the diverticulum area, was performed considering the influence of secondary SBV. **Non-specific inflammatory or neoplastic process with extensive mesenteric haziness with free fluid and a short segment of small bowel wall thickening in the mid-abdomen. ***Petersen’s defect was sutured closed.

No.	Author, year	Age, sex	Surgical history	CT findings	Preoperative diagnosis	Ischemia or necrosis	Bowel resection	Postoperative diagnosis	Surgical details
1	Sinicropi et al., 2024 [11]	41, M	MGBS	Whirl sign	SBV	None	None	SBV with CA	Detorsion
2	Sinicropi et al., 2024 [11]	83, M	None	Whirl sign	SBV	None	None	SBV with CA, diverticulum of the jejunum	Detorsion
3	Nakamura et al., 2024 [2]	93, M	None	Whirl sign	SBV	None	Partial*	SBV with CA, diverticulum of the jejunum	Detorsion, small bowel resection
4	Galvão et al., 2024 [5]	22, M	None	Whirl sign	SBV	Ischemia	None	SBV with CA	Detorsion
5	Hsu et al., 2024 [6]	37, F	PJBGC	Whirl sign	SBV	None	None	SBV with CA	Detorsion
6	Gupta and Mundasad, 2023 [3]	32, M	None	Internal hernia	Internal hernia	None	None	SBV with CA	Detorsion
7	Leaning, 2021 [7]	79, F	None	Whirl sign	SBV	None	None	SBV with CA	Detorsion
8	Vishnoi et al., 2019 [4]	80, M	LPEC	Non-specific**	Bowel ischemia	Ischemia	None	SBV with CA, omental bands	Detorsion band resection
9	Hayama et al., 2017 [8]	70, M	None	Whirl sign	SBV	None	None	SBV with CA	Detorsion
10	Akama et al., 2016 [9]	85, M	LDG	Whirl sign	SBV	None	None	Petersen hernia, SBV with CA	Detorsion suture closure***
11	Koh et al., 2013 [10]	19, M	None	Whirl sign	SBV	None	None	SBV with CA bands	Detorsion band resection
12	Our case, 2024	70, M	LTG	Edematous changes, ascites	SBV	None	None	SBV with CA	Detorsion

There was no anatomical abnormality that could have caused SBV in six out of 11 cases [3,5-8,11]. The body weight could not be confirmed, and not all cases had a history of surgery in the six cases. We assumed that dietary factors may also be the cause of SBV in those cases. Among the 11 previously reported cases, a case (9%) underwent small bowel resection because there was a diverticulum of the jejunum, considering the influence of secondary SBV [2]. However, no cases including our case required small bowel resection due to necrosis. We assumed that small bowel resection due to necrosis was not required in any case, as occluded lymphatic vessels led to small bowel and mesenteric edema, which inhibited further twisting. Additionally, given that SBV symptoms included acute abdominal pain, early surgical intervention was performed.

We did not perform preventative surgical measures for the recurrence of SBV, because the incidence of recurrence of adult SBV is generally low and there was no other evidence to justify preventative surgical measures [12]. Surgical prevention measures were also not performed in the 11 previously reported cases. We provided dietary guidance to prevent the recurrence of SBV because the cause of SBV in our case was strongly attributed to dietary factors in addition to low body weight following total gastrectomy.

## Conclusions

We experienced a case of acute small bowel obstruction secondary to SBV with CA following total gastrectomy. CPR is useful for diagnosing SBV in cases without typical CT findings. SBV with CA may not require small bowel resection due to necrosis, because occluded lymphatic vessels lead to small bowel and mesenteric edema, which inhibits further twisting. Additionally, surgical intervention is performed because of acute abdominal pain. We assume that dietary guidance is important because dietary factors may also be involved in SBV.
